# Successful use of stapokibart as a biological therapy for allergic fungal rhinosinusitis: a case report

**DOI:** 10.3389/falgy.2025.1746033

**Published:** 2026-01-14

**Authors:** Hua Cai, Xue-Jie Luo, Ya-Qi Cao, Sha-Zhou Li, Jian-Jun Chen, Liu-Qing Zhou, Tao Zhou

**Affiliations:** 1 Department of Otorhinolaryngology, Union Hospital, Tongji Medical College, Huazhong University of Science and Technology, Wuhan, Hubei, China; 2 Hubei Province Clinical Research Center for Deafness and Vertigo, Wuhan, China

**Keywords:** allergic fungal rhinosinusitis, biological therapy, case report, chronic rhinosinusitis, stapokibart

## Abstract

**Purpose:**

To present a rare, to our knowledge previously undescribed, case of successful AFRS management using stapokibart (CM310).

**Patients and methods:**

The laboratory, immunological, symptom scores and imaging datas before and after stapokibart treatment were evaluated to determine the effect of stapokibart on AFRS.

**Results:**

After four weeks of treatment, the patient showed significant clinical improvement, including a marked reduction in nasal polyp size and the near-complete return of normal olfaction. By week 16, lab tests confirmed a substantial drop in eosinophils and IgE, and a follow-up CT scan showed complete resolution of sinus inflammation. The patient reported high satisfaction due to a major improvement in quality of life.

**Conclusion:**

This pioneering case demonstrates that stapokibart, a novel anti-IL-4Rα antibody, is a promising and effective treatment for refractory AFRS, showing a rapid onset of action and sustained control of type 2 inflammation.

## Introduction

Allergic fungal rhinosinusitis (AFRS) typically presents at a younger age with more severe clinical manifestations, a higher recurrence rate, a pronounced impairment of olfactory function (1), and a strong association with environmental atopy. In light of the significant surgical burden and recurrence patterns, current research is evaluating biologics as a potential breakthrough in medical therapy (2). However, AFRS remains underrepresented in current research, highlighting a notable gap in focused investigation. In this letter, we present a rare, to our knowledge previously undescribed, case of successful AFRS management using stapokibart (CM310).

## Case report

We present the case of a 33-year-old male diagnosed with AFRS, who experienced recurrence of symptoms two months after undergoing endoscopic sinus surgery. Following recurrence, he was managed with intranasal corticosteroids but with inadequate response. He reported persistent nasal congestion, mucopurulent rhinorrhea, olfactory dysfunction, and headache, which significantly affected his daily life and work. Serum-specific IgE testing confirmed a high level of sensitization to a mold mix (3.7 KUA/L, Class 3—severe), which is pathognomonic for AFRS and consistent with the markedly elevated total IgE level of 399 KU/L observed at diagnosis. Nasal endoscopy revealed jelly-like secretions in the left middle meatus. Computed tomography demonstrated heterogeneous opacities (reflecting inspissated fungal mucin within hypodense mucosa), mucosal inflammation with sinus ostium obstruction, and bony remodeling evidenced by sinus expansion and focal erosions, the CT sinus scan was highly suggestive of AFRS. Histopathological examination revealed fungal elements, severe mixed inflammatory infiltrates, and abundant mucin deposition. Laboratory testing indicated an elevated eosinophil count of 0.65 × 10^9^ /L (reference range: 0.02–0.52 × 10^9^ /L) and a total IgE level of 399 KU/L (reference range: <60 KU/L).

Twelve months after the initial surgery, and following multidisciplinary evaluation, a therapeutic regimen was initiated, consisting of stapokibart 300 mg administered subcutaneously every two weeks in combination with intranasal corticosteroids. The treatment course lasted for 16 weeks, with routine monitoring of blood parameters, IgE levels, nasal endoscopy findings, and imaging outcomes.

## Results

**Figure 1:**
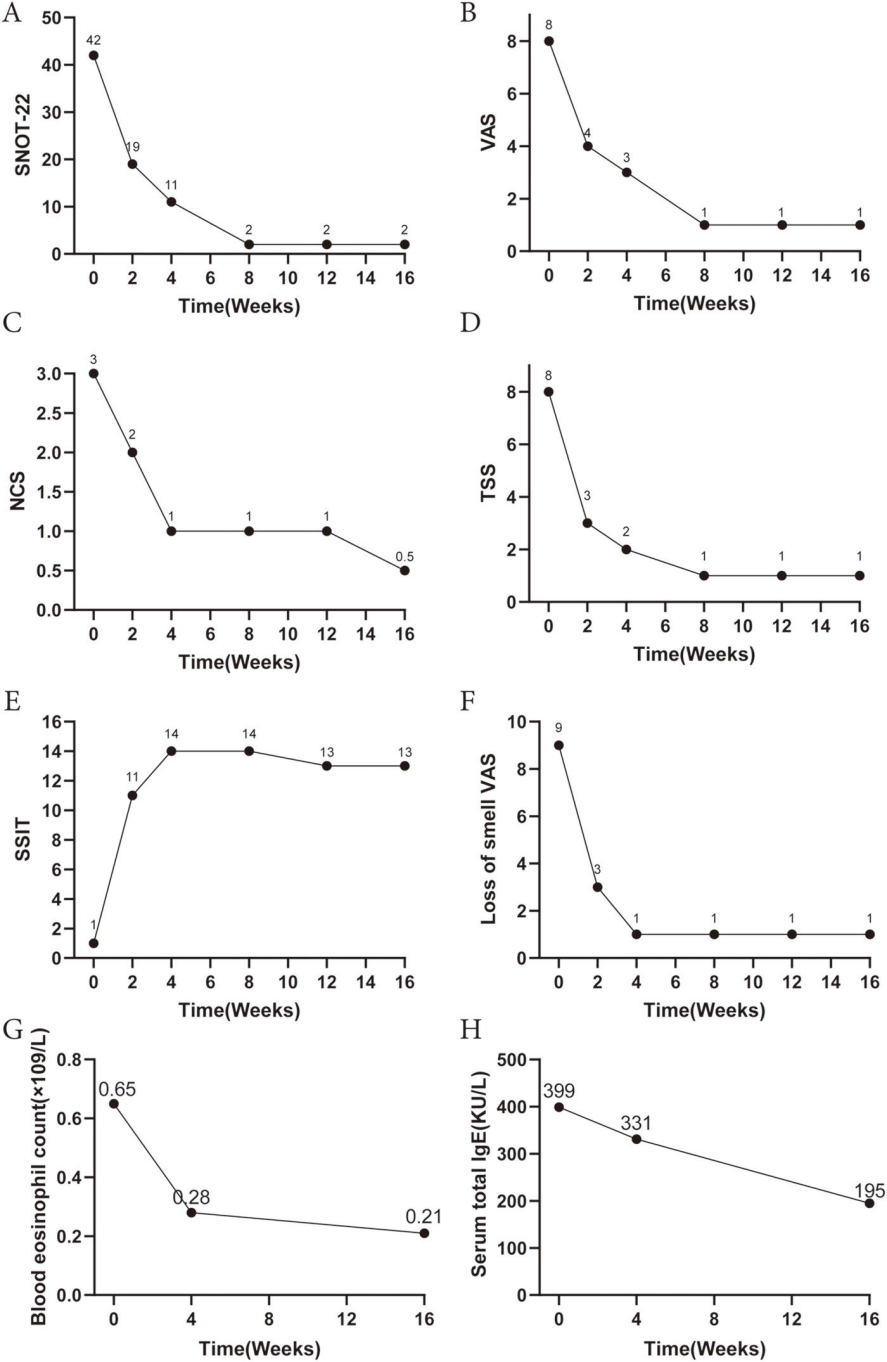
Laboratory, immunological and symptom scores before and after the administration of stapokibart. Comparison of **(A)** SNOT-22, **(B)** total nasal VAS score, **(C)** NCS, **(D)** TSS, **(E)** SSIT, **(F)** Loss of smell VAS scores, **(G)** Blood eosinophil count and **(H)** serum IgE level pre and post stapokibart therapy. SNOT-22, sinonasal outcome test-22; VAS, visual analogue scale; NCS, nasal congestion score; TSS, total symptom score; SSIT, sino-nasal symptom interview test.

**Figure 2:**
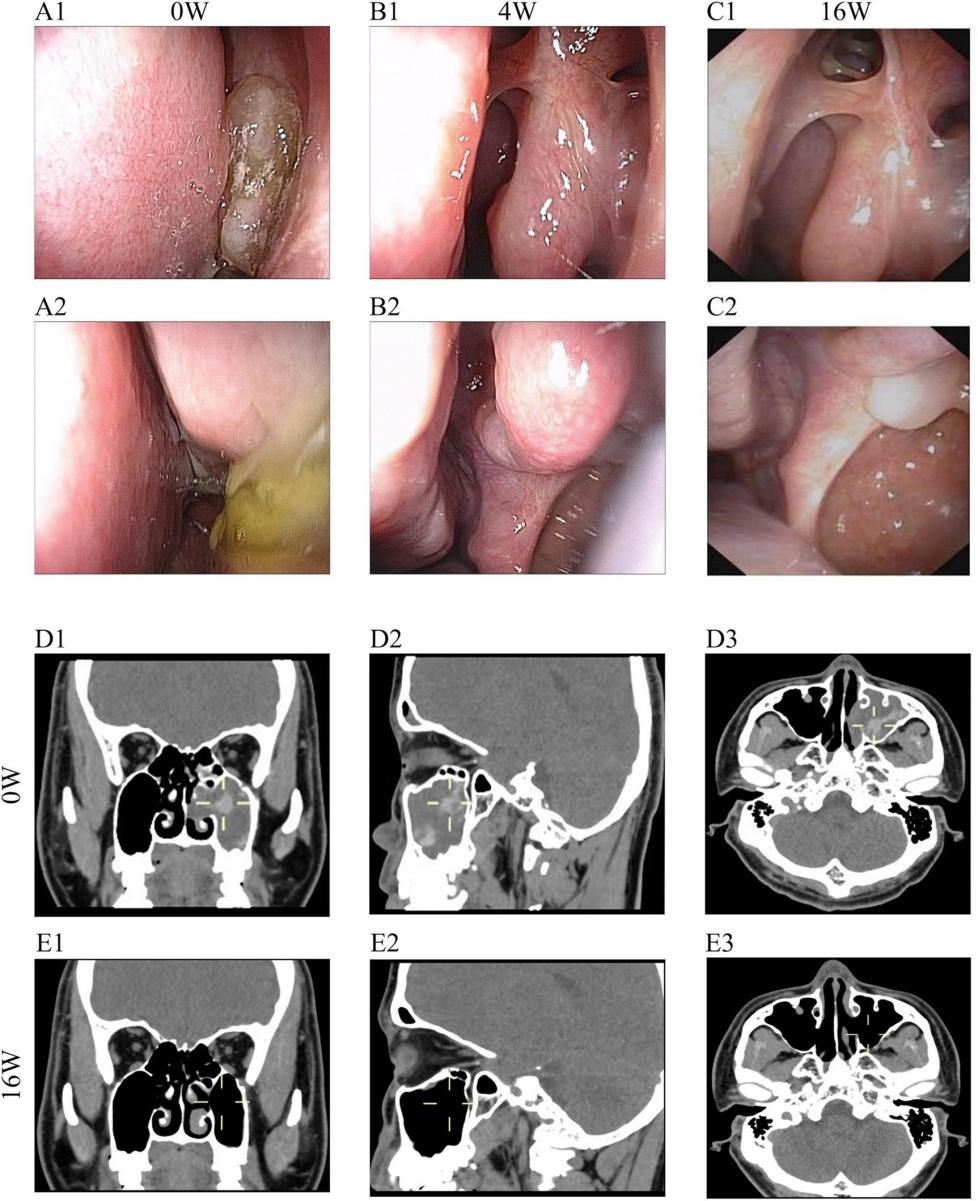
Imaging data of the case. **(A–C)** Comparison of endoscopic changes pre and post stapokibart therapy. (A1-A2) prior to treatment. (B1-B2) At 4-week follow-up after stapokibart therapy, endoscopic examination revealed complete resolution of the jam-like inspissated secretions in the left middle meatus. (C1-C2) 16 weeks after stapokibart therapy. **(D)** Pre-treatment CT scan of paranasal sinuses demonstrates unilateral, expansile lesions with heterogeneous opacities and noninvasive features. **(E)** 16 weeks after stapokibart therapy showed complete resolution of the inflammatory opacities in the left maxillary sinus and improvement of the ethmoid sinus mucosa.

After one week of treatment, the patient reported alleviation of nasal obstruction and discharge. By the second week, olfactory function began to recover. At week four, nasal endoscopy demonstrated marked reduction in polyp size, and olfaction had largely returned to normal. Corresponding to these clinical improvements, symptom scores showed substantial reductions: **the total nasal VAS score** decreased from 8 to 3, SNOT-22 from 42 to 11, nasal congestion score from 3 to 1, and total symptom score from 8 to 2 (4 weeks). Laboratory markers exhibited a progressive decline, with eosinophil count dropping to 0.28 × 10^9^ /L at week 4 and 0.21 × 10^9^ /L at week 16; total IgE decreased from 399 to 195 KU/L by week 16. Correspondingly, testing for a mold mix showed a level of 0.75 KUA/L at follow-up, corresponding to Class 2 (moderate) sensitization. (Figure 1). Follow-up CT at week 16 showed complete resolution of the inflammatory opacities in the left maxillary sinus and improvement of the ethmoid sinus mucosa (Figure 2). Olfactory testing confirmed complete functional recovery, with accurate identification and naming of all odors previously unrecognized. The patient expressed a high degree of satisfaction with the treatment outcomes, particularly noting the significant improvement in olfactory function and nasal airflow, which translated into a substantial enhancement in quality of life. In this case, stapokibart was well-tolerated over the 16-week treatment period with no adverse events reported. This observation is consistent with the favorable safety profile reported in prior clinical trials of stapokibart for related type 2 inflammatory conditions (3)**.** Provided are Pre- and Post-treatment nasal endoscopy images to visualize the progression (Figure 2).

## Discussion

The immunologic profile of AFRS closely parallels that of other CRSwNP subtypes, indicating that biologic therapy may serve as a feasible adjunctive treatment following functional endoscopic sinus surgery. Although biologic agents such as dupilumab, omalizumab, and mepolizumab have been approved for the treatment of CRSwNP, patients with AFRS were not included in these clinical trials (4–6).

Stapokibart (CM310) is a novel humanized IgG4 monoclonal antibody that specifically targets the interleukin-4 receptor alpha (IL-4Rα), which inhibits IL-4 and IL-13 signaling which underlying eosinophilic inflammation (7). Type 2 inflammation plays a central role in the pathogenesis of AFRS. Consequently, biologic agents that selectively inhibit key mediators of type 2 immune responses represent a promising therapeutic strategy for patients with AFRS. In clinical studies, Zhang et al. demonstrated that stapokibart showed favorable safety and significant efficacy in treating severe eosinophilic chronic rhinosinusitis with nasal polyps and seasonal allergic rhinitis (3, 8). While established biologics for CRSwNP such as dupilumab and omalizumab have demonstrated significant improvements in olfactory function over longer timelines (e.g., at 24 weeks) (2, 9), the rapid recovery observed in this case may be attributed to the potent and early reduction of mucosal edema and inflammation in the olfactory cleft facilitated by dual IL-4/IL-13 pathway inhibition. This pioneering case highlights stapokibart's potential as a novel therapy for refractory AFRS. Effects of dupilumab receded upon discontinuation at 24 weeks, suggesting that continuation may be needed for sustained disease control (4). Unlike dupilumab, stapokibart interacts with M39, S95, and L135 and binds to IL-4Rα closer to the ligand binding site (10), suggesting distinct mechanisms and clinical outcomes. Notably, a marked reduction in peripheral eosinophil count and total serum IgE levels was observed, indicating effective suppression of the underlying immunologic drivers of AFRS.

## Conclusion

These findings highlight the therapeutic potential and rapid onset of action of stapokibart in refractory AFRS, demonstrating symptomatic and pathological improvement. Optimal duration of stapokibart in AFRS patients is unknown. Further clinical trials are required to confirm efficacy. Critically, long-term monitoring is essential to assess durability, monitor for potential recurrence or contralateral spread, and identify predictive biomarkers. Future studies should also explore its role within comprehensive strategies that may include specific immunotherapy.

## Data Availability

The raw data supporting the conclusions of this article will be made available by the authors, without undue reservation.
